# Development of the Carers’ Alert Thermometer (CAT) to identify family carers struggling with caring for someone dying at home: a mixed method consensus study

**DOI:** 10.1186/s12904-015-0010-6

**Published:** 2015-05-03

**Authors:** Katherine Knighting, Mary R O’Brien, Brenda Roe, Rob Gandy, Mari Lloyd-Williams, Mike Nolan, Barbara A Jack

**Affiliations:** Evidence-Based Practice Research Centre, Edge Hill University, Faculty of Health & Social Care, St Helens Road, Ormskirk, Lancashire L39 4QP UK; Liverpool Business School, Liverpool John Moores University, Liverpool, L3 2AJ UK; Academic Palliative and Supportive Care Studies Group, Institute of Psychology, Health and Society, University of Liverpool, Liverpool, L69 3GL UK; The School of Nursing & Midwifery, University of Sheffield, 3a Clarkehouse Road, Sheffield, S10 2LA UK

**Keywords:** Carers, Caregivers, End-of-life care, Home care, Needs assessment, Palliative care

## Abstract

**Background:**

There is an increasing international policy direction to promote home death for dying patients which will impact on the demands placed on family carers. The early identification of carer needs and appropriate intervention can help avoid crisis situations for the carer and avoidable hospital admissions which are reported to be a global concern. The aim of the study was to explore what professionals and carers of patients with cancer and advanced progressive illness, in their last year of life, find burdensome and to develop an alert system for use by non-specialist staff.

**Methods:**

A mixed-method, multi-phased, consensus study sequentially utilising qualitative and quantitative data to develop and pilot the Carers’ Alert Thermometer (CAT). 245 people (117 carers and 128 professionals) participated in the study across a range of health and social care settings in the North West of England (2011–2014).

**Results:**

A number of key domains were identified and prioritised by consensus for inclusion in the CAT. The 8 domains fit within two overarching themes of the reported carer experience; the support needed by the carer to provide care and the support needed for the carer’s own health and well-being. The resultant CAT is an evidence-based alert thermometer consisting of 10 questions, guidance on the possible actions for each alert and space for an action plan to be jointly agreed by the assessor and carer. Preliminary piloting of the CAT has shown it to be valued, fit for purpose and it can be administered by a range of personnel.

**Conclusions:**

The CAT enables the identification of current and potential future needs so a proactive approach can be taken to supporting the carer as their role develops over time, with a view to enhancing their well-being and preventing avoidable hospital admissions; ultimately supporting patient choice to remain in their own home.

## Background

Globally approximately 50% of deaths occur in the hospital setting, with a wide variation ranging from 78% in Japan to 20% in China, resulting from a variety of societal and cultural factors [1]. Within the United Kingdom (UK), home deaths fell from 31% in 1974 to 18% in 2003, with approximately 60% of people dying in hospital each year; around 40% of hospital deaths result from advanced non-curative disease [2,3]. Despite this figure, patients, the public and health professionals identify being cared for at home, and dying there, as their preferred choice [2,4,5Several countries, including the UK and New Zealand, have tried to redress this trend for hospital death with various policy initiatives, such as innovative models of hospice to home care [2,6-8]. In the UK there are early indications of a reversal in the tendency towards hospital deaths [4]. However, despite policy initiatives, services are not universally available or provided in a timely manner [9]. Therefore, caring for a dying person at home relies heavily upon the support of informal, or family, carers. For the purpose of this study we have adopted the broad definition used by the Social Care Institute for Excellence (SCIE) that states: we define family carer ‟to mean a person or people identified by the person dying (where possible) as important to them, and it is intended to cover a spouse, partner, child, other relative, friend or supporter who cares for, and cares about, the person who is dying. Where a person is dying at home, the primary meaning of “carer” is a person who delivers everyday care to the dying person” [9].

With an increasing ageing population, the number of people undertaking the role of family carer is estimated to rise. Currently in the UK a conservative estimate is that 6.5 million people are acting as family carers, of which approximately 500,000 are caring for someone in the last year of life [10]. A recent study by Carduff *et al*., [11] highlighted the ‘hidden carers’ who are not known to health and social care services, partly due to them not identifying themselves as carers, thus adding to the estimated figure. Furthermore, this figure comprises a growing ageing population with the over-65 year old population accounting for 17% of the total, and an increasing proportion of ‘older old’ carers aged 85+ [12]. This is coupled with the changing nature of family and household composition. An increasing divorce rate, geographical mobility, including European migration and immigration, all result in potential family carers being dispersed or having to care for family members not resident in the same home or even geographical area. Similarly, the reduction of the post war baby boomer generation, increasing number of women who work, and a rise in the pensionable retirement age, can result in increased pressure on a smaller number of family carers who may themselves be older and therefore more frail and vulnerable [11,13]. In addition to these growing societal challenges for family carers, there is the emerging ‘sandwich generation’ where carers may have multiple caring roles for parents along with children or grandchildren [2,14]. A population study by Burns *et al*. in Australia identified a large ‘invisible network’ of carers who provide support to those dying at home but remain unrecognised by health services as they are not identified next-of-kin family members [15]. Better methods of identifying and supporting all types of carers are crucial if their vital role is to be sustained and people supported to die at home [15]. Recognising carers’ needs and understanding the type and level of support available to them is vitally important if the goal of achieving a good home death is to be realised [16]. Supporting carers in their caring role is a key policy in many countries; the appreciation of carers as partners or co-workers in providing care is recognised within the End-of-Life Care Strategy in the UK [7], the need to identify and address carers’ needs is also evident in Canada [17], Australia [18] and within Europe [19]. In the UK the need to support carers has been further highlighted within the recently released priorities for care of the dying person issued by the Leadership Alliance for the Care of Dying People [20]. Priority four states that ‘The needs of families and others identified as important to the dying person are actively explored, respected and met as far as possible’ (p87).

### Carer strain and burden

A two-part evidence review of published research (1998–2008) of home-based family caregiving at the end-of-life, reported the strain that caring can bring [21,22]. This strain can encompass psychological difficulties including anxiety, depression and fear of not coping. Physical challenges are inherent in the provision of bodily care, for example helping with mobility, but also encompass the carer’s own physical needs, particularly those of elderly carers. Disturbed sleep and the resulting fatigue for the carer materialise as the disease progresses. Carers can also experience increasing social isolation as more care is required and a lack of normality in their lives, particularly those with multiple caring roles [9]. Lastly, there are financial consequences of caring including an impact on employment [21,22]. Recently Gardiner *et al*. reporting on the economic burden of caring, highlighted the changing trajectory of caregiving particularly towards the end-of-life, with intense periods of caring developing [23]. They propose there is a need for the ongoing assessment of family carers, with early initiated financial support to reduce carer burden and potentially prevent hospital admission [23]. Family carer breakdown has been reported to contribute to inappropriate hospital emergency admissions [6,24]. A study from New Zealand, reported physicians referring to admission for ‘compassionate reasons’ or complex social admissions due to caregiver burden as ‘all of a sudden the caregiver, usually the spouse, cannot cope it just gets too hard’ [6] (pg 6). Gott *et al*. propose the term ‘potentially avoidable’ for these hospitalisations if appropriate support for carers is provided [6]. Carer strain is typically cumulative with a final, often seemingly insignificant, issue being too much for the carer to cope with, when it is too late for supportive interventions to be put in place [6]. Supporting family carers was also noted in a retrospective study of general practitioners in The Netherlands, when asked their views on the avoidance of hospitalisation at the end-of-life [25].

Although carer assessment tools are available, ranging from lengthy research instruments to ad-hoc service developed tools; a systematic review of 62 instruments identified no evidence-based tools for the assessment of carers providing end-of-life care in the home, which were fit for everyday use in practice [26]. Many tools were found to measure carer satisfaction with the care provided, quality of life and family functioning. Six tools were identified with a primary focus on caregiver burden and five on caregiver needs. These tools covered a variety of domains including psychological well-being, social support and impact on the carer’s lifestyle. Some were multidimensional and the average number of items was 25. Reliability and validity were only reported for a limited number of tools (e.g. The Caregiving at Life’s End questionnaire [27] and The General Family Functioning Scale of the Family Assessment Device [28]). Since the review the Carers Support Needs Assessment tool (CSNAT) has been developed [29]. In contrast with the tool reported in this paper, the CSNAT was developed with bereaved carers of patients receiving support from specialist palliative care via hospice home care services [29]. The CSNAT consists of 14 broad domains including physical, social, financial and spiritual support needs which are rated on a four point scale and used to develop a shared action plan. No data have been reported about completion times required for the tool. The CSNAT has been validated with adult carers [30] and implementation has been conducted with carers of patients receiving hospice home care [31]. Feedback demonstrated the value of assessing carer needs but significant challenges to implementation on individual and organisation levels have been reported even within specialist palliative care home-care services [32]. The challenge of implementing carer assessments within the specialist services who are viewed as providing a high standard of holistic care is concerning, particularly as the majority of patients with advanced, progressive illness do not receive specialist palliative care. Normally, they are cared for by community health care teams, which may include health care assistants (support staff who are not on the professional nursing register) who do not have the time or expertise to undertake a comprehensive carer assessment. Additionally, there is widespread use of private agencies providing care support in the community both for long-term conditions and advanced progressive disease, with the majority of their staff being care assistants. For carers’ needs to be assessed regularly any tool used must be fit for purpose, by the staff that have the most frequent contact with carers. There is a need for a tool that is quick and easy to use which can act as an alert to proceed to a comprehensive formal assessment to be undertaken by experienced health or social care professionals. Drawing upon the concept of the modified early warning systems (MEWS) which detect early signs that patients are in need of a higher level of care [33], we developed the Carers’ Alert Thermometer (CAT) that can be used in daily practice in the home, by non-specialist staff, to identify carers who are at risk and in need of a formal needs assessment. This paper discusses the findings of a multi-phase study which explored what professionals and carers of patients with cancer and advanced progressive illness, in their last year of life, find burdensome and the development an alert system for use by non-specialist staff to trigger provision of appropriate support, referral to services or further in-depth formal assessment.

## Methods

The CAT was developed using a mixed method consensus design which collected, analysed and integrated qualitative and quantitative data sequentially across five phases between 2011 and 2014 in North West England [34]. Item generation was conducted using semi-structured interviews and focus groups with carers. Item selection was achieved by consensus using a two-round Delphi survey with carers, health and social care professionals and voluntary sector staff. This method has been extensively used within health care research for the development of clinical guidelines and identification of priorities for development [35,36]. The Delphi technique uses a structured approach with a series of questionnaires (or rounds) with ‘experts’ which are continued until consensus is reached. Its strength lies in the premise that group opinion has greater validity than individual opinion [37]. A third-round consensus with an expert panel refined the CAT items which was then piloted in different health settings. The pilot findings were then reviewed in a consultation exercise with a range of professionals and carers. All stages of the study were approved by the Faculty of Health and Social Care Research Ethics Committee at the University and Local Research Ethics Committees. Informed consent was obtained from all participants throughout the study. A summary of the aim, design, data collected and participants across the five phases is outlined in Table 1. The method and results for each phase are outlined together in the results section due to the iterative nature of the study.

**Table 1:** **Overview of study design and participants**

**Phase**	**Aim**	**Design & data type**	**Participants**
**Phase 1a**	To capture carers’ experiences, identify factors causing stresses/burdens during the caring experience and views on the use of a carer’s alert tool	Prospective semi-structured interview study with carers (qualitative data)	18 carers (14 current carers and 4 carers who were bereaved during the study)
Months 1-16			
**Phase 1b**	Same as Phase 1a	Focus group study with carers (qualitative data)	5 focus groups at carer centres and a hospice involving 25 carers (19 current carers and 6 bereaved carers)
Months 1-16			
**Phase 2**	To gain consensus on the most important factors to be included from Phase 1 for inclusion in the CAT	A two round Delphi survey (quantitative and qualitative data)	151 surveys were completed across the two rounds by 126 participants.
Months 17-18		Round 1: 44 items across 8 topic domains	Round 1 = 43 professionals, 42 carers
		Round 2: 29 items across 8 topic domains	Round 2 = 44 professional, 22 carers
			Professionals were from charities, carer’s centres, university, hospices, NHS Trusts (Primary Care, Community Care, Hospitals), local authority and personal social services (n = 81)
			Carers were current and bereaved carers (n = 45)
**Phase 3**	To seek expert panel review of the top ranked factors of carer burden from Phase 2, and consensus selection of the final list of 10 items for the pilot CAT	Consultation and consensus selection (quantitative and qualitative data)	6 professionals from national and regional organisations with a strategic role in End of Life care and carer support and 4 carers who participated in Phase 1 and 2 of the study.
Month 19			
**Phase 4**	To pilot the readability and usability of the initial CAT	Pilot study of the initial CAT (quantitative and qualitative data)	8 professionals across 4 clinical sites:
Month 20–24 (Official end of study)			• 4 District Nurses across two Community NHS Trusts
			• A Clinical Services Manager at an adult hospice,
			• Two Clinical Nurse Specialists and a Social Worker at a tertiary cancer centre.
			7 current carers across the sites
**Phase 5**	To review the findings of the pilot and consult with potential user groups on any revisions to be made to the CAT tool	Consultation review meetings with potential users (quantitative and qualitative data)	• 18 carers at a carers centre
Months 30-35			• Consultation meetings with lead professionals at the pilot clinical sites and with professionals attending a national consultation day organised by a national nursing organisation. Professionals included Palliative Care Consultants, District Nurses and Team Managers, Community Matrons, NHS Commissioners, Nurse Educator, Chief Executive of a national nursing organisation, and Managers of voluntary organisations supporting carers (n = 33).

Total number of participants in the study = 245.

## Results

### Phase 1: item generation

#### Recruitment

Phase 1 participants were identified by general practitioners (GPs) or through advertising the project with local support groups, carer centres and adult hospices and local media coverage across the North West of England as well as via the project website. Potential participants were sent a recruitment pack containing study information and contact details for the research team. All recruited carers were over 18 years of age and either caring for someone in their expected last year of life or bereaved after a period of providing care.

Of the 43 carers who participated in Phase 1, 26 (60%) were female and all, except one, were related to the care recipient with 27 (64%) being a spouse and 13 (30%) being adult children. Carers were aged between 20 and 80 years old. Fourteen (33%) care recipients had a primary diagnosis of cancer; the remainder had other advanced progressive conditions such as respiratory or neurodegenerative diseases or dementia.

#### Procedure

The majority of the 18 interviews were conducted within the home of the carer or patient, with a small number taking place at an adult hospice. Three focus groups were conducted with 25 carers in carer centres and an adult hospice. A semi-structured guide was used for interviews and focus groups (see Table 2). Carers were provided with information about local services and sources of support after data collection. All data were digitally recorded and transcribed verbatim.

**Table 2:** **Phase 1 topics and questions in the semi-structured guide**

**1. Demographic information**	a) Age
	b) Employment status – current, previous, reasons for stopping work, how long ago stopped work
	c) Relationship with person currently caring for
	d) Any previous caring roles
**2. Perception of carers**	a) *Would you describe yourself as a carer*?
	b) If not, how *do you see your role as being different to that of a carer*?
**3. Current caring role**	*a*) *How long have you looked after* ………….?
	*b*) *Can you describe some of the things that you do for*…? (physical, social, psychological)
	*c*) *Looking back at the time you have been caring for* …., *have you seen a change in how you care for* ….?
	(Changes, increases in care required)
	d) *Have there been any occasions when you have found caring to be challenging*? (examples & context)
	*e*) *Looking back on the* …. *months that you have been caring for* …., *what were the most challenging things you* *have had to deal with*, *and why*?
	f) *Is it difficult to say that there have been problems or challenges*? *Or to ask for help*?
	g) *What helps you to cope with the challenges*? (what helps most, least)
	h) *Is there anything positive that has come out of your caring role with* ….?
**4. Support and assessments**	a) *What support or help have you received whilst caring for* …?
	b) *Is there any help or support you feel you might have benefited from but did not receive*?
	*c*) *Have you had any assessments since you started caring for* ….?
	*d*) *Did those assessments result in any additional support*?
	*e*) *Do you think regular assessment of carers needs would be helpful*?
	*f*) *Are there any areas in particular that you feel should be assessed*?
	g) *How would you feel being asked about your needs on a regular basis by someone who comes into your home* *regularly*, *such as a district nurse*? (Any other professional you would prefer?)
**5. Open ended question**	*Is there anything else you*’*d like to add to what you*’*ve said today*?

#### Analysis

Adopting a thematic analysis approach [38], two researchers independently analysed a proportion of the data identifying a list of emergent themes which were then discussed, refined and agreed before being applied to the full dataset for Phase 1. The key themes and factors identified were further reviewed with the study steering group, including carer representatives, to inform the item development for the Delphi survey. Two main overarching themes were identified in the data which spanned the domains and factors; the support needed by the carer to provide care and the support needed for the carer’s own health and well-being.

### Phase 2: item selection

#### Delphi development

The themed factors carers identified as causing stress or burden in Phase 1 were developed into a two-round Delphi survey, thus allowing for the provision of feedback and the opportunity to revise earlier responses [36]. An inclusive approach was taken for Round 1 resulting in eight domains with 44 items. A table of the domains with a definition, example and number of items for each domain from the survey is provided (Table 3).

**Table 3:** **Domain themes and survey examples**

**Domain**	**Qualitative data summary**	**Survey item example**	**Number of items**
		“*How important is it to assess*…..”	
**1. Understanding the current caring context**	Carers spoke about the importance of understanding their ‘lived situation’ such as who they were caring for, other demands on their time, and their understanding of the diagnosis and prognosis of the person they were caring for	…if the carer understands the expected progress of the condition of the person they are caring for?	10
**2. Current care provided by the carer**	Carers were providing many different levels of care including physical, emotional and practical care	…if the carer feels able to support the emotional needs of the person they care for?	4
**3. Carer’s relationship with professionals**	Carers spoke about their relationships with multiple professionals who were providing care or treatment to the person they cared for, whether they felt excluded or included in discussions about the care of the person they cared for and about their relationships with professionals supporting their own health and social care.	… if the carer feels that professionals involve them in decision making by seeking their knowledge and expertise about the care needed by the person they care for?	4
**4. Respite and emergency care support**	Carers spoke about the need for a break or respite care and their concerns about what would happen to the person they care for in an emergency or if they were unable to provide care	… if the carer has planned what should happen in an emergency if they were unable to provide care, e.g. if they become ill or go into hospital?	3
**5. Financial support and assessments**	Carers spoke of the stress caused by financial issues and the lack of systematic assessment or support for carers generally	…if the carer knows of and has applied for all appropriate funding, such as benefits, mobility schemes?	7
**6. Carer’s own health and well-being**	Carers tended to put their own needs after those of the person they care for but many carers had their own health concerns and some spoke of the importance of needing time for themselves.	… if the carer is able to balance their own health needs with the demands of caring?	6
**7. Support for the carer**	Carers spoke of ‘not knowing what support was available’ until they met a ‘*gateway*’ person who provided information or access to services.	… if the carer has received information about the carer support available in their area?	6
**8. End of Life (EoL) Care and planning**	Carers spoke of needing to focus on the current caring situation rather than EoL planning but recognised the importance of knowing the person’s wishes	… if the carer knows the wishes and preferences of the person they care for, and they have been written down and shared, e.g. advance care planning (ACP) document?	4

The Delphi survey was created online using SurveyMonkey® and was also available in paper format. It was aimed at family carers and a range of professionals who support carers and patients during their end-of-life care. The purpose was to rate the importance of the items which arose from Phase 1 and begin the process of reaching consensus on the priority items to be included in the CAT. A small pilot study was conducted (n = 5) and minor modifications made based on feedback received.

There is no consensus in the literature as to the appropriate number of subjects to involve in a Delphi study [39]. The important factor is the selection of subjects to ensure the most informed experts are selected. A purposive sampling approach was taken to recruit community-based health and social care professionals and staff who work closely with carers in the voluntary sector in the North West of England. All invitees were asked to cascade the email invitation to other relevant professionals as appropriate. Carers who had participated in Phase 1 were also invited to take part. Fliers advertising the survey were distributed to carer centres, support groups, other voluntary organisations and adult hospices in the region, including those who had facilitated carer recruitment in Phase 1.

#### Round 1

The Round 1 survey contained three sections. In *Section A* participants were asked to rate the importance of each of the 44 items for inclusion in the CAT using a 5 point Likert scale from 1 ‘not at all important’ to 5 ‘extremely important’. A comment box was also provided for each domain. In *Section B* participants were asked their views on the development and future use of the CAT and to rank the eight topic domains in order of preference for inclusion in the CAT. In *Section C* participants provided anonymous demographic information about themselves and their caring or professional experience. A final section contained useful contacts for carers including the local carer centres.

In Round 1, there were 85 participants (see Table 1 for details). Data were entered into IBM SPSS Statistics for Windows (Version 20.0. Armonk, NY: IBM Corp.) for analysis. Descriptive statistics were used to explore the mean, median and standard deviation to identify the ratings of both professional and carer cohorts and the total sample for individual items and for the ranking of domains [40]. The level of consensus to include an item in the CAT was set at 70% of participants (in each cohort or in the total sample) rating the item as equal to, or greater than a mean level of 4 (very important). The level of consensus within each cohort and the total sample was assessed by reviewing the frequencies for each item to see the percentage of the total sample who rated the items at the level of 4 and above. Open text comments from professionals and carers were subject to thematic analysis approach as in Phase 1.

Analysis of the Round 1 responses showed a high level of consensus on the importance of the items for inclusion in the CAT. The mean ratings for 34 of the 44 items was equal to or above the mean level of 4 (very important) with a consensus level of 70% or above. Due to the high level of rating and consensus across the items it was decided that a total sample median of 5 (extremely important) would be used to determine the items to be included in the subsequent round, in addition to any items where there was disagreement between the cohorts; consequently 29 items were included in Round 2.

#### Round 2

The Round 2 survey also contained three sections. Given the high level of rating in Round 1 and the reduced number of items, *Section A* became a ranking exercise for participants to prioritise their responses to identify the key items for inclusion in the CAT. Participants were asked to rank the items in each domain from 1 (most important item) to the least important until they had ranked all items in each domain; the number of items in each domain ranged from 2 to 6. They were provided with the group summary response from Round 1 and asked to rank the eight topic domains for a second time. Those who had not taken part in Round 1 were asked to complete the original *Section B* and *Section C* from Round 1 to provide demographic data and their views on the future CAT.

There were 66 participants in Round 2. Again, there was a high level of agreement with both cohorts ranking the same item as the most important in each of the seven domains. There was disagreement in the end-of-life care and planning domain as carers ranked an item related to carer bereavement support as ‘most important’ whereas professionals ranked the carer knowing the wishes and preferences of the patient and having the appropriate documentation completed as the ‘most important’. Where there was disagreement the total sample means were used to identify the priority and the items referred to the expert panel.

Both professional and carer cohorts consistently ranked ‘financial support’ and ‘end-of-life care and planning’ domains as lower priorities than understanding the ‘current caring situation’ and the ‘carer’s health and well-being’ domains (see Table 4).

**Table 4:** **Ranking of domains in Round 1 and Round 2**

**Domain**	**Round 1 ranking**	**Round 2 ranking**
**Domain 1** Understanding the current caring situation	**1st**	**1st**
**Domain 2** Current care provided by the carer	4th	4th
**Domain 3** Carer’s relationship with professionals	5th	5th
**Domain 4** Respite and emergency care needs	6th	6th
**Domain 5** Financial support and assessments	7th	7th
**Domain 6** The carer’s health and well-being	**2nd**	**2nd**
**Domain 7** Support for the carer	3rd	3rd
**Domain 8** End of life care and planning	**8th**	**8th**

### Phase 3: expert panel consultation

Further refinement of the items to be included in the CAT was achieved following consensus by an expert panel. This comprised of six representatives from national and regional organisations, including leaders from a leading UK hospice and a UK national nursing organisation with a strategic role in end-of-life care and carer support, and four carers who had participated in earlier phases of the study.

The panel were asked to select their top 10 items from the 16 highest ranked items across the eight domains identified in Round 2 and provide comments or highlight any issues they felt were missing. Qualitative analysis was performed on the panels’ comments using thematic analysis [38]. Panel feedback was very positive about the comprehensiveness of the items; most said it was challenging to reduce the items as all were viewed as important.

“*I feel that this is a comprehensive list of the issues that are important to carers*” (*Professional*, *P3 panel*).

“*I found this quite hard to choose a top ten because if an important element of the support is missing it has a domino effect on the quality of support the carer can give the patient. Most carers will put their needs bottom of the list and some will not seek carer support in their area*.” (*Carer*, *P3 panel*)

The top 10 items ranked for inclusion were subject to the same analytic process as Phase 2, to identify consensus within both professional and carer cohorts and across the total sample. Although there was a wide spread of rating on some items, reflecting the individual nature of this type of assessment, overall, there was good agreement across the expert panel. Table 5 shows the group means for the top ranked 10 items in Phase 3.

**Table 5:** **Top 10 ranked items for inclusion in the CAT following Phase 3 expert panel (n = 10)**

**Domain theme**	**Item**	**Ranking**	Mean ranking* (SD)
D1: Understanding the current caring situation	…if the carer understands the expected progress of the condition of the person they are caring for?	1	2.88 (2.64)
D2: Current care provided by the carer	… if the carer feels able to support the psychological/emotional needs of the person they care for?	2	3.25 (3.86)
D3: Carer’s relationship with professionals	… if the carer feels that professionals involve them in decision making by seeking their knowledge and expertise about the care needed by the person they care for?	3	3.88 (1.55)
D4: Respite and emergency care needs	… if the carer would like support with a break from caring such as using a sitting service in their home for a few hours or to use respite care for a longer break? (if services available)	4	4.00 (2.12)
D3: Carer’s relationship with professionals	… if the carer feels they are receiving the support they need from professionals at the time they need it?	5	4.13 (2.17)
D2: Current care provided by the carer	… if the carer has a named person or number to call in an emergency or with any concerns about the person they care for?	6	4.50 (2.98)
D1: Understanding the current caring situation	… if the carer has responsibility for making decisions about the care of the person they care for, due to their condition or mental capacity?	7	5.20 (3.27)
D5: Financial support and assessments	… if the carer knows of and has applied for all appropriate funding, such as benefits, mobility schemes?	8	5.86 (1.86)
D7: Support for the carer	… if the carer feels they are currently receiving enough support?	9	6.00 (3.35)
D6: The carer’s health and well-being	… if the carer is able to balance their own health needs with the demands of caring?	10	6.11 (3.41)

*Items were ranked from ‘1’ as the highest ranked item so the items ranked highest have the lowest mean.

It is interesting to note that six of the top ten items were from domains 1 (Understanding the current caring situation), domain 2 (Current care provided by the carer), domain 3 (Carer’s relationship with professionals) and domain 4 (Respite and emergency care needs), one item each from domains 5 (Financial support and assessment), 6 (Carer’s health and well-being), and 7 (Support for the carer), and none from domain 8 (End-of-life care and planning).

### Phase 4 & 5: CAT pilot and consultations

The CAT was developed containing 10 ‘alert’ questions to assess the highest ranked factors of carer burden, with an additional question from the end-of-life domain which was appropriate to include given the population that the CAT will be used with. The total number of alerts identified for each carer was marked on a thermometer scale to indicate their level of need. Space was provided to note the frequency of review required and a plan of action for that individual carer that would be develop jointly by the assessor and the carer. Guidance on possible action to be taken for each alert was also provided, although this did not replace the staff member’s professional responsibility for taking appropriate action.

### Rigour

A small validity pilot was conducted to test the readability and usability of the CAT for staff and carers with eight health professionals from community healthcare trusts, an adult hospice and a tertiary cancer centre and seven family carers. Opinions were gathered using feedback forms from professionals and telephone interviews with carers. Consultation exercises with 18 carers and 33 health professionals helped to further refine the CAT.

## Results of the pilot and consultations

The CAT was well received by both carers and professionals. Feedback indicated the CAT was comprehensive, enabled discussion about issues of concern to the carer, and would be a valuable tool to identify current as well as future areas of carer burden.

The clear accompanying instructions and visual appearance of the CAT were liked by carers and professionals who also felt the thermometer scale provided a clear indicator of the carer’s overall level of need. Furthermore, the action plan was viewed as an obvious reminder of what had been agreed and a means of tracking progress. During the pilot, the CAT successfully identified a number of alerts. Consequent actions included the provision of, or signposting to, information and referral for packages of care to improve the support going into the home. The typical completion time for the first administration was approximately 20 minutes.

Participants recommended simplification of some of the wording used to avoid possible misinterpretation and provision of additional space to document any issues raised and actions to be taken. To help prioritise the response needed to any identified alerts and promote agreed understanding between the assessor and the carer it was suggested that an indicator of risk be included for each item.

### Recommendations on implementing the CAT

Both carers and professionals recommended early and repeated use of the CAT to regularly review carers’ needs, especially when providing end-of-life care at home. It was seen as applicable to a wide range of staff, but crucially, for continuity, the CAT should be completed by someone with whom the carer has an ongoing relationship. Additionally, carers suggested that staff administering the CAT should provide written information about locally-available services and support for carers.

A cautionary note was made by carers and professionals, that asking questions on specific topics could raise carers’ expectations that particular support was available when this might not be the case everywhere. To avoid unrealistic expectations, both groups suggested starting with an open question with a checklist of prompts or follow up questions to enable carers to talk about what was important to them whilst serving as a reminder to staff of key areas to be covered in the discussion.

### Summary of the CAT

Throughout the development of the CAT there was an overwhelming response, by both current and past carers, as to the value of a regular assessment. This value of assessment is reflected in the UK Leadership Alliance for the Care of Dying People report as being essential to underpin the priority of supporting family carers [20]. Carers in the study highlighted that many of them struggle with the caring role and particularly in accessing appropriate information and signposting for support services. This finding is not unexpected and is reflected in a recent initiative by hospice and palliative care services which, having recognised the issue, have introduced volunteers to support carers in the process, for example the Marie Curie Helper Service [41].

The overall findings from this study are supported by the available research indicating that carers neglect to identify themselves as carers or being in need of support. Referred to by Harding and Higginson [42] as ‘ambivalent care’, rather than considering their own needs, central to carer concerns is being able to focus on providing the best possible physical and emotional care. The need for carers to be fully informed of the patient’s condition and what was likely to happen as the illness progressed was also a main focus; direct support for themselves was consistently underplayed, which is congruent with the literature [11,29].

In response to the feedback from the pilot and consultations some changes were made to the CAT appearance and wording of questions. The CAT is presented with two main themes: the ‘*current caring situation*’ which assesses any support needs the carer has providing care in the current situation. This theme covers physical, psychological and information needs including the availability of an emergency support contact and ascertaining whether financial and legal advice is needed. The inclusion of financial assessment is supported by the recent study by Gardiner *et al*. which suggests this as a possible contributory factor in family carer breakdown [23]. Additionally, spiritual care needs are included which not only reflect the growing emphasis on holistic assessment but also the possibility that the need for spiritual support could facilitate a discussion to identify other areas of carer need [43]. The second theme of ‘*carer*’*s health and well*-*being*’ assesses any support needs the carer has for themselves. The literature indicates that carers neglect to consider their own needs [42] so asking questions specifically to assess the carer’s needs may encourage them to actually consider their own needs and help with anticipating increasing health issues.

End-of-life planning was ranked the lowest by both carers and health care professionals. This was an unexpected finding, especially from the health care professionals, in light of the introduction of policy initiatives in the UK supporting end-of-life and advance care planning [7]. It could be suggested this is due to a lack of confidence amongst health care professionals who may not have had communication skills training to undertake these conversations [44]. The low priority for end-of-life planning may also be influenced by the strong focus of carers and professionals on the current situation and meeting those demands rather than looking too far ahead. However, consultation with the expert panel confirmed the importance of the topic and the need for early planning of end-of-life preferences making it appropriate for this issue to be included on the CAT to ensure it is addressed in a timely manner for the patient and carer. Therefore we have included a final question on end-of-life care planning, to be asked if appropriate. This reflects the increasing promotion of end-of-life discussion by organisations such as Dying Matters, a UK national coalition, which aims to change public knowledge and attitudes to death and dying by starting to undo the taboo subject of death and promote conversations around end-of-life matters [45]. Furthermore, the need for communication around dying is also central to recently released priorities for end-of-life care in the UK [20].

The final CAT therefore contains ten questions across the two themes (see Table 6 for questions). Each question has a traffic light system of green (Low), amber (Intermediate) and red (High) so the carer and the assessor can discuss the situation and tick the level of risk each alert poses to the caring situation or the carer. The three levels of risk, as described above, have been indicated in the traffic light images with the initials L, I and H to support black and white printing where needed. The total number of intermediate and high risk alerts is then noted on the thermometer providing a visual overview of the areas which need monitoring or an immediate response. Guidance is provided to signpost the assessor to what actions may be taken to address the alerts which can be adapted to meet local service provision and avoid raising expectations where services may not be available. There is also space for the assessor to write a plan of action for the priority alerts, which have been agreed with the carer, to facilitate follow up and monitoring of any actions required. For example, question 3 is “*Do you need any help to provide any of the physical or general daily care your* [*person*] *requires*?” Where a carer has no current need a shared decision may be to tick the green ‘low’ risk light on this occasion. If a carer was struggling with physical care, and there was a perceived risk to the carer or care of the patient, the decision may be to tick the red ‘high’ risk light indicating the need for immediate action. This alert would be included in the total alerts on the thermometer and identified in the action plan with actions such as referral for equipment or additional help coming into the home, along with a nominated person who is responsible for the action and follow up with the carer. The CAT is available for dissemination and use with supporting guidance available at the project website [46].

**Table 6:** **Questions from Section 2 of the CAT**

**Discuss the following areas with the carer to identify any alerts requiring action.**	
[x] = person being cared for e.g. husband or wife.	
**(A) CURRENT CARING SITUATION**	
**Q1**	Do you have any needs or concerns about caring for your [x]?
**Q2**	Do you need any information about the condition your [x] has and how the care needed might change over time?
**Q3**	Do you need any help to provide any of the physical or general daily care your [x] requires?
**Q4**	Do you need any help to provide any emotional or spiritual care your [x] requires?
**Q5**	Do you have a named person to call in an emergency or out-of-hours to discuss any concerns about your [x]?
**(B) CARER’S HEALTH AND WELL-BEING**	
**Q6**	Do you feel involved in discussions and listened to by professionals about the care needed by [x]?
**Q7**	Do you need any help or information about money or legal issues?
**Q8**	Do you need a break from caring during the day or overnight?
**Q9**	Do you need any help to balance your own needs with the demands of caring? (*e.g. attend own health appointments*, *social activities*)
**Q10**	If appropriate include: Do you know your [x]’s wishes and preferences for EoL care? (If known, have they been written down and shared, e.g. advance care planning (ACP) doc?)

## Discussion

This paper has reported on the development of an alert indicator to identify family carers of patients with advanced and progressive disease who are in their last year of life, who may be at risk of increased strain and have unidentified needs. The CAT was developed using a multi-phased approach capturing the views of current carers, past carers, professionals from health and social care, and the voluntary sector and policy advisors. This wide range of participants has enabled a breadth of experiences to be drawn upon and makes the development process of the CAT distinct from other carer assessments which have relied primarily on data from one source, typically bereaved carers or professionals, or established scales which have been adapted. Importantly, endorsement by the expert panel ensures that the CAT addresses issues that have been identified at national policy level.

The increasing international policy direction to promote home death, along with the development of end-of-life care in resource poor countries, will undoubtedly have an impact on the demands placed upon family carers [16]. This policy direction is coupled with an increasing ageing population, the emerging ‘sandwich generation’ of carers, and geographical mobility; factors which will result in a smaller pool of family carers, many of whom will be ageing and with increasingly complex caring responsibilities [2,11,14]. Carers’ needs will change, particularly across the illness trajectory; early assessment and appropriate intervention can help to avoid crisis situations which can lead to carers collapse, and prevent avoidable hospital admissions that are reported to be an issue of concern globally [6,25].

It is widely reported that carer strain is cumulative [6], especially when caring for someone in the final months of life [47], and it is often one final issue, however seemingly small, which takes the family carer from a position of coping to one of crisis. The findings from this study suggest that there is a need for a proactive approach to regular carer assessments, preferably by someone who has a relationship with them. The CAT’s design is unique amongst carer assessment tools as it based on the principle of the MEWS alert system [33], facilitating a focused appraisal of how a carer is coping in key areas which are then individually risk assessed and provide a visual assessment to show if carer needs are increasing over time. The CAT can then be followed up with appropriate targeted interventions and, if needed, a full and detailed assessment on any aspect of need. Preliminary piloting of the CAT has shown it to be valuable and it can be administered by a range of health and social care professionals, including non-registered staff. Further research is undoubtedly required, and the next stage is a feasibility study with carers from a range of settings. Since its launch in Autumn 2014 the CAT has been adopted by various NHS and charity organisations and it is intended that an initial feasibility study will explore issues of implementation, including training needs and use by health care assistants and other unregistered staff. Consideration will be given to any training and administration differences for registered and unregistered staff. The planned study will enable the CAT to be administered over a longer period of time with carers, enabling the team to follow alert changes and the actions taken to support the carer, in addition to conducting validity test-retest and reliability scale coefficient analysis. Carers who participated in the study have also raised the potential for self-reporting by carers either on paper or electronic forms such as a smart phone app and the use of it by staff in carer centres. As with any new systems, consideration for the appropriate training and support of all personnel who could administer the CAT will be explored in future studies. The CAT offers a short proactive alert tool to identify carers with unmet needs, which may help to support the carer in their role and own well-being, and prevent ‘potentially avoidable’ hospital admission whilst supporting patient choice to remain in their own home.

### Limitations

The study faced a number of recruitment challenges. Firstly, a period of intense change within the UK health care system at that time resulted in a lack of engagement by clinical staff. This was addressed by amending the recruitment strategy. Secondly, the intended recruitment of carers through primary care health services proved difficult and may well have been a consequence of the lack of carer identification as noted by Carduff *et al*. in their reference to ‘hidden carers’ [11]. Additional methods of recruitment were incorporated to overcome this problem by drawing on local networks already established with the research team.

The study was mainly conducted in North West England which limits the findings, however including representatives from national organisations within an expert panel strengthens the findings and increases their transferability. Additionally, the findings are reflective of the wider national and international literature.

However, a particular strength of this study is the involvement of carers in the design and process of the study and in the development of the CAT, ensuring that it is based on priorities identified by carers. The study has included both current and bereaved carers providing valuable insight into their support needs.

## Conclusions

The CAT is an evidence-based alert thermometer consisting of 10 prioritised questions to assess carers’ need for support across two overarching themes of the support needed by the carer to provide care and the support needed for the carer’s own health and well-being. The CAT enables the identification of current needs and potential future needs so a proactive approach can be taken to support the carer as their role develops over time, ultimately enabling them to maintain their well-being and to provide end-of-life care at home. Although developed in the UK, the CAT has the potential to be adopted internationally and across a range of other long term conditions and settings.
